# Changing sustainable diet behaviours during the COVID-19 Pandemic: inequitable outcomes across a sociodemographically diverse sample of adults

**DOI:** 10.1017/jns.2024.9

**Published:** 2024-03-14

**Authors:** Elizabeth Ludwig-Borycz, Ana Baylin, Andrew D. Jones, Allison Webster, Anne Elise Stratton, Katherine W. Bauer

**Affiliations:** 1 Department of Nutritional Sciences, University of Michigan, Ann Arbor, USA; 2 Department of Epidemiology, University of Michigan, Ann Arbor, USA; 3 International Food Information Council Foundation; 4 Department of Sustainable Use of Natural Resources, University of Hohenheim

**Keywords:** COVID-19 pandemic, equity, food environment, nutrition, sustainable diets

## Abstract

The objective of this study was to describe changes in sustainable dietary behaviours (those that support environmental, economic, and physical health) among a sample of US adults during the COVID-19 pandemic and to examine differences in changes by individuals’ race/ethnicity and socioeconomic status. Therefore, a cross-sectional online survey study was conducted in April 2021 (N = 1,488, mean age = 42.7 (SD = 12.6)) receiving outpatient care from Michigan Medicine, the University of Michigan health system. Enrolment quotas were established to ensure a diverse sample—one-third of participants identified as African American/Black, one-third Hispanic/Latino, one-third White, and one-third low-income. Participants reported engaging in more behaviours that are supportive of a sustainable diet one year into the COVID-19 pandemic compared to before. This is particularly true regarding ecologically and economically sustaining behaviours such as taking fewer trips to the grocery store, increased use of home grocery delivery, increased cooking at home, and greater consumption of healthy foods. Not all behaviour changes promoted sustainable food systems; namely, the use of farmer’s markets and Community Supported Agriculture (CSAs) declined. White and high-income participants were more likely than African American/Black, Hispanic/Latino, and low-income individuals to engage in ecologically and economically sustainable dietary behaviours during the pandemic. Meanwhile, African American/Black participants reported large increases in physical health sustainable dietary behaviours. To support the continuation of greater engagement with sustainable diets, policies that increase access to public transportation, limit the frequency with which consumers have groceries delivered, increase work-from-home options, and improve access for low-income populations should be prioritised.

## Introduction

In March 2020, the novel coronavirus SARS-CoV-2 (COVID-19) spread across the US, growing into a pandemic that infected nearly 100 million and killed over a million people within 2 years.^(1)^ To help contain the virus, local governments quickly implemented restrictions on citizens to support social distancing including quarantining at home, closing non-essential in-person businesses and schools, and restricting social gatherings. As such, many lost their jobs and 2020 became a year of record-high unemployment and food insecurity.^(2,3)^ By early 2021, unemployment numbers had improved but were still well above pre-pandemic rates by about 3%.^(4)^ These conditions were inequitably distributed, disproportionately affecting Black/African American, Hispanic/Latino, and low-income families, and may have contributed to poor diet quality.^(4,5)^ Overall, these alterations led to rapid changes in how individuals engaged with the food environment and the context of their dietary behaviours.

The majority of research regarding COVID-19 pandemic-related dietary changes has been conducted from the lens of physical healthfulness. For example, studies conducted early in the pandemic identified that consumption of fruit, vegetables, alcohol, and sweets increased during the early months of the pandemic as compared to pre-pandemic, while meat consumption decreased.^(6,7)^ Additionally, Americans shopped less frequently in person, relied on online shopping to avoid in-person interactions, and cooked more often at home versus purchasing fast food, takeout, or ready-made meals.^(7,8)^ However, changes in consumer behaviour also have implications for the sustainability of the food system.^(9)^ Sustainable diets are those that promote ecological, economic, human, socio-cultural, political health, and well-being.^(9)^ Downs et al.^(9)^ embeds sustainable diets into the food environment framework, describing the ecological and economic dimensions as supporting agricultural production systems that promote biodiversity, local and seasonal foods; conserve soil and water; lower GHGE; and minimise food loss and waste. The human health dimension is defined as supporting the thriving of human health and well-being through plant-based, nutrient-dense foods that meet macro- and micro-nutrient requirements. The EAT-Lancet Planetary Health Diet (PHD) is one way to measure the human health dimension of a sustainable diet.^(10)^ The PHD was created in 2019 to optimise human health by ensuring a diet that meets nutritional adequacy (both macro- and micro), is primarily plant-based and nutrient-dense, along with operating within safe planetary boundaries (GHGE, nitrogen, phosphorus, water use, biodiversity loss, and land use).^(10)^ Examples of dietary behaviours that are, on average, more sustainable (including ecological, economic, and human health dimensions) include minimising grocery store trips,^(11–15)^ shopping for locally grown produce and other food,^(9)^ shopping at a farmer’s market or participating in a CSA (Community Supported Agriculture) subscription,^(9)^ growing a vegetable garden or participating in a community garden,^(9)^ making more foods from scratch, and decreasing food waste.^(16,17)^ Finally, the socio-cultural and political dimension examines issues of equity and disparities within the food system.^(9)^

The US is the 2^nd^ highest emitter of carbon dioxide globally and food systems account for approximately one-third of GHGEs.^(18,19)^ Improving the sustainability of Americans’ dietary behaviours can help combat climate change while at the same time, improving the nutritional status of Americans.^(9)^ Behaviours such as minimising animal-sourced food consumption^(20)^ and decreasing food loss and waste^(16,21)^ have the potential to reduce food-related emissions by up to 50% globally. Understanding how US consumers’ behaviours changed during the first year of the pandemic can illuminate key areas of food environment change that can continue to be supported to help consumers maintain these behaviours. Conversely, identifying how dietary behaviours became less supportive of a sustainable diet during the pandemic can provide insight into areas of the food environment in need of further investment, policy, and structural change.

The objective of this study is to describe changes in dietary behaviours among a sociodemographically diverse sample of US adults from the perspective of joint ecological, economic, human health, socio-cultural, and political sustainability one year into the COVID-19 pandemic as compared to before the pandemic. Further, we will examine differences in changes in these behaviours by individuals’ race/ethnicity and socioeconomic status in order to better understand equity in opportunities for sustainable dietary behaviours during the pandemic. Through the aforementioned changes to consumer behaviour, we hypothesise that behaviours will have become, on average, better aligned with sustainable diets during the COVID-19 pandemic compared to before the pandemic. This knowledge will inform policy initiatives, with specific attention to the needs of underserved populations, that can support the continuation of sustainability-promoting behaviours that increased during the COVID-19 pandemic and better promote sustainable behaviours that did not change or worsened during the pandemic.

## Methods

### Study population

Data were obtained from SUSTAIN, an online survey conducted in April 2021. Potential participants were identified through a query of adult patients (age range: 18-65) who received outpatient care from Michigan Medicine, the University of Michigan’s health system, between March 2019 and March 2020. To ensure racial, ethnic, and socioeconomic diversity of the study sample, enrolment quotas were established to enforce that one-third of participants identified as African American/Black, one-third Hispanic/Latino, and one-third White. Additionally, enrolment limits were established to ensure that at least one-third of participants were low-income, defined as being insured by public insurance (Medicaid). To accomplish these goals, all patients aged 18-65 who identified as Black/African American (10,547 with public insurance and 15,307 with private insurance) or Hispanic/Latino (2,918 with public insurance and 8,139 with private insurance), and had an email in their electronic health record, were emailed an invitation to participate in the study. Due to the large number of patients identifying as White (253,462), 10,547 White patients with private insurance and 15,307 White patients with public insurance were randomly selected to receive a study invitation. The participant invitation described the study as seeking to learn more about people’s food choices during the COVID-19 pandemic and included a unique link to a Qualtrics-based eligibility screening survey.^(22)^

The screening survey identified individuals who were, (1) living in the state of Michigan since at least March 2020; (2) involved in food choices/shopping for their household; (3) ages 18 through 65 years old; and (4) fluent in English. Of the 2,625 participants who completed the screening survey (response rate 4.2%), 2,439 (92.9%) were eligible to participate, and 1,488 completed the study survey. Although eligible, the remaining 951 individuals completed the screening survey after enrolment limits had already been met, and therefore did not continue to the study survey. See Supplemental Figure 1 for a flow chart of participant enrolment. Study participants who completed at least 85% of the survey questions received the opportunity to enter a lottery for 1 of 10, $100 gift cards as compensation for their participation. This study was conducted according to the guidelines laid down in the Declaration of Helsinki and all procedures involving human subjects/patients were approved by the University of Michigan Institutional Review Board (HUM: 00191932). Written informed consent was obtained from all subjects/patients.

### Survey development

Development of the study survey was based on sustainability attributes of foods and beverages using the food environment framework created by Downs et al.^(9)^ The food environment framework outlines dimensions of sustainable diets: ecological and economic, human health, and socio-cultural and political. Survey questions that aligned with the ecological, economic, and human health dimensions were selected from existing measures of consumer behaviour and dietary intake, as well as surveys of the food environment conducted during the COVID-19 pandemic.^(23)^ Selected questions were then tested for comprehension and applicability via cognitive interviews among a sociodemographically diverse sample of adults (n = 20) and modified based on participant feedback.^(24)^

### Study measures

#### Ecological and economic sustainable diet behaviours

To assess this dimension, participants were asked, “Please indicate how much on average you and your household have done the following?, (1) over the past year during the COVID-19 pandemic (defined as March 2020 to the present - at the time of survey administration) and (2) before the COVID-19 pandemic (defined as before March 2020)^(25)^ for each of the behaviours listed in Supplemental Table 1. Response options were on a 5-point Likert or frequency scale, tailored to the item being assessed. Driving to the grocery store, eating from restaurants, and eating pre-packaged/ready-made meals were reverse coded, and responses to all 13 items were then summed to create an ecological and economic dimension score ranging from 0 to 52 for the two different time points, where higher values represent greater sustainability within the dimension. A change in the ecological and economic dimension score was also calculated by subtracting the over the past year score from the before COVID-19 pandemic score.

#### Human health sustainable diet behaviours

Alignment of participants’ diets with the human health dimension of sustainable diets was assessed using the EAT-Lancet Planetary Health Diet (PHD) food group categories.^(10)^ Participants were asked, “Please indicate how much on average you have eaten the following foods over the past year during the COVID-19 pandemic (since March 2020) compared to before COVID-19 (before March 2020). I eat ________ now than I did before the COVID pandemic.” for the following food groups: vegetables, fruit, potatoes, whole grains, dairy, eggs, poultry, meat, fish, soy, nuts, legumes, and sweets. Response options were on a 5-point Likert scale, “a lot more; more; the same amount; less; a lot less.” Responses to the questions about beef, lamb, or pork; potatoes; dairy; sweets; eggs; chicken and other poultry were reverse coded and then responses to all questions were assigned a point value from -2 (“a lot less”) to 2 (“a lot more”). Scores across the 13 items were summed,^(26)^ to create a human health dimension score ranging from -26 to 26, with higher positive values representing greater sustainability.

#### Socio-cultural and political sustainable diet dimensions

Insight into the socio-cultural and political dimension of sustainable diets was gleaned by examining differences across the other dimensions with regard to socioeconomic status and race/ethnicity, thereby allowing us to understand potential disparities. Sociodemographic information including participants’ race/ethnicity (White, Black or African American, Hispanic or Latino, Asian American, American Indian or Native American, and Other), income, and number of household members was collected in accordance with the 2020 US Census and National Health and Nutrition Examination Survey (NHANES) questions.^(27,28)^ Participants that identified as more than one race/ethnicity were categorised in accordance with The National Longitudinal Study of Adolescent to Adult Health.^(29)^ If participants self-identified as Hispanic or Latino then that participant was given a race designation of “Hispanic” and eliminated from the other marked categories; the process was repeated in the following order: Black or African American, Asian American, American Indian or Native American, Other, and White.^(29)^ After the descriptive analysis only participants who were categorised as White, Black or African American, or Hispanic or Latino were included (Supplemental Figure 1). Income to needs ratio (ITN) was calculated using participants’ household income and total household size in accordance with the US Department of Health and Human Services poverty guidelines for 2021 and then stratified into tertiles.^(30)^

#### Covariates

Age and gender were collected using 2020 US Census questions.^(27)^ Household food security was assessed using the six-item short form of the Food Security Survey recommended by the United States Department of Agriculture.^(31)^

### Statistical analysis

Descriptive statistics were calculated for participant age, gender, household food security, race/ethnicity, and ITN. The mean ecological and economic dimension score, change in mean ecological and economic dimension score, and change in human health dimension, along with their individual components, were also calculated. Analysis of covariance, ANCOVA, was used to examine differences in continuous variables (mean ecological and economic dimension score, change in mean ecological and economic dimension score, change in human health dimension, along with their individual components) by race/ethnicity and adjusted for education, gender, and ITN. ANCOVA was also used to examine differences in continuous variables (mean ecological and economic dimension score, change in mean ecological and economic dimension score, change in human health dimension, along with their individual components) by ITN and adjusted for education, gender, and race/ethnicity. If overall tests indicated differences in behaviours by race/ethnicity or ITN, pairwise comparisons between means for racial/ethnic or ITN categories were examined using Tukey’s Studentized Range. The alpha level at which differences were considered significant was less than 0.05. Statistical analyses were carried out in SAS version 9.4.

#### Statistical Power and Sample Size

A minimum sample size of 300 per group was selected to detect small to moderate size differences (d = 0.162) in sustainable diets before the COVID-19 pandemic and during the COVID-19 pandemic between key sociodemographic groups participating in the survey at alpha = 0.05 and 80% power.

## Results

Study participants (n = 1,488) mean age was 42.7 (SD = 12.6) years old with 77.4% of participants identifying as female, 22.0% as male, and 0.5% as another gender (Table 1). Race/ethnicity of the study sample was equally distributed between White, Black/African American, and Hispanic/Latino with 18 participants identifying as other races/ethnicities. More than half (53.9%) of participants reported having a bachelor’s degree or higher. When disaggregated by race/ethnicity, 23.9% of Hispanic or Latino, 42.4% of Black or African American, and 36.6% of White individuals were low-income.

**Table 1.** Sociodemographic characteristics of sustaining participants (n = 1,488)

	Mean (SD) or % (N)
Age (years)	42.7 (12.6)
Gender	
Male	22.0 (328)
Female	77.4 (1152)
Other	0.5 (8)
Race/Ethnicity	
White	36.3 (540)
Black/African American	33.6 (500)
Hispanic/Latino	28.9 (430)
Asian America	0.2 (3)
American Indian or Native American	0.3 (4)
Other	0.7 (11)
Educational attainment	
Some high schools or less	2.9 (41)
Finished high school or got GED	7.7 (108)
Did some college or training after high school	22.3 (314)
Associate’s degree or completed technical training	13.3 (187)
Bachelor’s degree	27.5 (387)
Advanced degree (Master’s, Ph.D., MD)	26.4 (372)
Household income (ITN)	
Low (0.1-1.9)	34.9 (476)
Moderate (2.0-4.4)	31.6 (431)
High (4.8-11.6)	33.5 (457)
Household food security	
High or marginal food security	70.6 (879)
Low food security	17.3 (215)
Very low food security	12.1 (151)

### Ecological and economic dimension

Participants’ ecological and economic sustainable diet scores during the COVID-19 pandemic in comparison to before the COVID-19 pandemic are shown in Table 2. Overall, participants’ ecological and economic sustainable diet scores improved during the COVID-19 pandemic compared to pre-pandemic (mean effect size = 1.2, *p* < .0001). Improvements were achieved through large decreases in eating at a restaurant either indoors or outdoors (effect size = −1.0, *p* < .0001), decreases in driving to the grocery store (effect size = −0.6, *p* < .0001), increases in eating foods that were traditionally purchased pre-made but now made at home (effect size = 0.4, *p* < .0001), and increases in eating meals that were home-cooked (effect size = 0.3, *p* < .0001). However, not all changes to the dimension were improvements; we observed declines in shopping at a farmer’s market or participating in a CSA (effect size = −0.6, *p* < .0001) along with shopping for locally grown produce and/or other food (effect size = −0.3, *p* < .0001).

**Table 2.** Ecological and economic sustainable diet behaviours before and during the COVID-19 pandemic (n = 1,488)

	Before COVID	During COVID	Cohen’s d	P-value^a^
	Mean (SD)			
Shop for locally grown produce and/or other food^b^	1.9 (1.1)	1.6 (1.1)	−0.3	<0.0001
Shop at a farmer’s market or participate in a CSA^b^	1.5 (1.1)	0.9 (1.0)	−0.6	<0.0001
Grow your own produce^b^	0.9 (1.2)	0.9 (1.2)	−0.0	0.68
Drive to the grocery store^c^	1.8 (1.4)	1.1 (1.1)	−0.6	<0.0001
Have groceries delivered to your home^c^	0.1 (0.6)	0.3 (0.6)	0.2	<0.0001
Use a meal kit delivery^c^	0.2 (0.9)	0.4 (1.2)	0.1	0.0003
Eat foods that are traditionally purchased pre-made but someone made at home^d^	1.6 (1.1)	1.7 (1.2)	0.4	<0.0001
Eat something from a fast-food restaurant^c^	2.7 (2.0)	2.4 (1.9)	−0.1	<0.0001
Eat takeout or delivery from a restaurant^c^	2.3 (1.8)	2.3 (1.6)	0.0	0.45
Eat at a restaurant either indoors or outdoors dining^c^	2.5 (1.4)	0.8 (1.3)	−1.0	<0.0001
Eat meals that are home-cooked^c^	8.4 (5.0)	9.4 (5.1)	0.3	<0.0001
Eat pre-packaged meals such as frozen dinners, canned soup, or ramen noodles^c^	2.4 (2.8)	2.7 (3.0)	0.2	<0.0001
Throw food away^e^	2.5 (0.9)	2.6 (0.9)	0.1	<0.0001
Ecological & economic dimension score (0 to 52)	23.4 (5.9)	24.5 (6.2)	1.2	<0.0001

a Paired t-test comparing before and during COVID; Cohen’s d calculating effect size.

b 0-4, where 4 = Always and 0 = Never.

c Times per week.

d 0-4, where 0 = Always and 4 = Never.

e 0-4, where 0 = A great deal and 4 = None at all.

### Human health dimension

On average, alignment of participants’ diets with the PHD improved during the COVID-19 pandemic as compared to before the pandemic (Table 3). Specifically, using a scale of −2 (less sustainable) to 2 (more sustainable) to quantify the degree of change from before COVID, participants reported eating more vegetables (mean = 0.4 (SD = 1.0)), fruit (mean = 0.4 ((SD = 1.0)), and nuts (mean = 0.2 (SD = 0.9)) during COVID, as well as less beef, lamb, and pork (mean = 0.2 (SD = 0.9)). However, participants also reported eating less soy food (mean = −0.3 (SD = 1.0)) and more poultry (mean = −0.3 (SD = 0.9)) and eggs (mean = -0.2 (SD = 0.9)).

**Table 3.** Changes in human health sustainable diet behaviours before versus during the COVID-19 pandemic (n = 1,051)

	Mean (SD)^a^
Vegetables^b^	0.4 (1.0)
Fruit^b^	0.4 (1.0)
Nuts^b^	0.2 (0.9)
Beef, lamb, or pork^c^	0.2 (0.9)
Potatoes^c^	0.1 (1.0)
Fish^b^	0.1 (1.0)
Beans, lentils, or peas^b^	0.1 (0.9)
Dairy^c^	0.1 (0.9)
Whole grains^b^	0.1 (0.8)
Sweets^c^	−0.1 (1.1)
Eggs^c^	−0.2 (0.9)
Chicken and other poultry^c^	−0.3 (0.9)
Soy food (including tofu and soy milk)^b^	−0.3 (1.0)
Human health dimension score (-26 to 26)	0.7 (4.9)

a Positive values are more sustainable and negative values are less sustainable according to the PHD.

b -2 = A lot less and 2 = A lot more.

c -2 = A lot more and 2 = A lot less.

### Socio-cultural and political dimension

On average, high-income households and White participants had a higher ecological and economic dimension score for sustainable diets both before and during the COVID-19 pandemic compared to lower-income and Black/African American and Hispanic/Latino participants (Table 4). Individual ecological and economic sustainable diet behaviours varied considerably by income and race/ethnicity. Before COVID, high-income participants more frequently ate at restaurants either indoors or outdoors (2.7 (0.2)), compared to low (2.2 (0.2)) and moderate (2.5 (0.2)) income participants (*p* < .0001) (Table 5). However, during the COVID-19 pandemic, a large decline in eating at restaurants was seen among all income groups, with the largest declines among high-income households (low −1.3 (0.2), moderate −1.6 (0.2), and high −1.9 (0.2) (*p* < .0001)). This decline was countered by an increase in eating meals prepared at home, again with the largest change being for high-income households (low 0.4 (0.5), moderate 0.8 (0.5), and high 1.2 (0.5) (*p* = 0.0001)). Additionally, high-income households increased the amount of food traditionally purchased pre-made that they now made at home (0.3 (0.1)) more than low (0.1 (0.1)) or moderate (0.1 (0.1)) income households (*p* < .0001). Differences in consumption of pre-packaged meals such as frozen dinners, canned soup, or ramen noodles did not vary by income category. Before COVID, White participants more frequently shopped for locally grown produce (2.3 (0.2)), compared to Hispanic or Latino (2.1 (0.2)) and Black or African American (1.9 (0.2)) participants (*p* < .0001). Additionally, White participants more frequently shopped at farmer’s markets or participated in a CSA (1.9 (0.2)), compared to Hispanic or Latino (1.6 (0.2)) and Black or African American (1.5 (0.2)) participants (*p* < .0001). During COVID, a large decline was seen among all racial/ethnic groups for shopping for locally grown produce (White −0.6 (0.1), Hispanic or Latino −0.5 (0.1), and Black or African American −0.4 (0.1) (*p* = .009)) and shopping at farmer’s markets or participating in a CSA (White −1.0 (0.2), Hispanic or Latino −0.8 (0.2), and Black or African American −0.7 (0.2) (*p* = .0002)), with the largest declines among White participants.

**Table 4.** Sustainable diets before and during the COVID-19 pandemic by household income and race/ethnicity (n = 1,346)

	Household income^1^			P-value	Race/Ethnicity^2^			P-value
	Low (mean (standard error))	Moderate (mean (standard error))	High (mean (standard error))		Hispanic/Latino (mean (standard error))	Black/ African American (mean (standard error))	White (mean (standard error))	
Ecological and Economic Dimension Before COVID-19	22.8 (0.8)^a^	23.4 (0.9)^b^	24.3 (0.9)^c^	<0.0001	23.2 (0.9)^a^	22.5 (0.8)^b^	24.8 (0.8)^c^	<0.0001
Ecological and Economic Dimension Change during COVID-19	1.0 (0.5)^a^	1.2 (0.5)^ab^	1.6 (0.5)^b^	0.0009	1.4 (0.5)^a^	1.5 (0.5)^a^	0.9 (0.5)^ab^	0.006
Human Health Dimension Change during COVID-19	0.4 (0.8)^a^	0.6 (0.8)^ab^	0.9 (0.8)^b^	0.03	0.3 (0.8)^a^	1.6 (0.8)^b^	0.0 (0.8)^a^	0.0002

*Note*: Sustainable diet means with common superscript letters did not differ at *p* < 0.05 using Tukey’s Studentized Range.

1 Adjusted for education, race, and gender.

2 Adjusted for education, ITN, and gender.

**Table 5.** Associations between income, race/ethnicity, and ecological and economic dimension (n = 1,346)

	Household income^1^			P-value	Race/Ethnicity^2^			P-value
	Low	Moderate	High		Hispanic or Latino	Black or African American	White	
	Mean (SD)				Mean (SD)			
Shop for locally grown produce and/or other food^3^								
Before COVID	2.0 (0.2)^a^	2.1 (0.2)^a^	2.2 (0.2)^a^	0.07	2.1 (0.2)^a^	1.9 (0.2)^a^	2.3 (0.2)^b^	<0.0001
Change during COVID	−0.5 (0.1)^a^	−0.5 (0.1)^a^	−0.4 (0.1)^a^	0.35	−0.5 (0.1)^ab^	−0.4 (0.1)^a^	−0.6 (0.1)^b^	0.009
Shop at a farmer’s market or participate in a CSA^3^								
Before COVID	1.5 (0.2)^a^	1.7 (0.2)^ab^	1.8 (0.2)^b^	0.03	1.6 (0.2)^a^	1.5 (0.2)^a^	1.9 (0.2)^b^	<0.0001
Change during COVID	−0.8 (0.2)^a^	−0.9 (0.2)^a^	−0.9 (0.2)^a^	0.34	−0.8 (0.2)^a^	−0.7 (0.2)^a^	−1.0 (0.2)^b^	0.0002
Grow your own produce^3^								
Before COVID	0.8 (0.2)^a^	0.9 (0.2)^a^	0.9 (0.2)^a^	0.35	0.8 (0.2)^a^	0.6 (0.2)^b^	1.2 (0.2)^c^	<0.0001
Change during COVID	0.1 (0.1)^a^	0.1 (0.1)^a^	0.2 (0.1)^a^	0.11	0.2 (0.1)^a^	0.2 (0.1)^a^	0.1 (0.1)^a^	0.08
Drive to the grocery store^4^								
Before COVID	1.5 (0.2)^a^	1.4 (0.2)^a^	1.5 (0.2)^a^	0.41	1.3 (0.2)^a^	1.4 (0.2)^a^	1.7 (0.2)^b^	<0.0001
Change during COVID	−0.7 (0.2)^a^	−0.6 (0.2)^a^	−0.6 (0.2)^a^	0.74	−0.6 (0.2)^a^	−0.5 (0.2)^a^	−0.7 (0.2)^a^	0.04
Have groceries delivered to your home^4^								
Before COVID	0.1 (0.1)^a^	0.1 (0.1)^a^	0.0 (0.1)^a^	0.20	0.0 (0.1)^a^	0.1 (0.1)^b^	0.0 (0.1)^a^	0.002
Change during COVID	0.2 (0.1)^a^	0.1 (0.1)^a^	0.1 (0.1)^a^	0.75	0.1 (0.1)^a^	0.1 (0.1)^a^	0.2 (0.1)^a^	0.69
Use a meal kit delivery^4^								
Before COVID	0.1 (0.1)^a^	0.1 (0.1)^ab^	0.2 (0.1)^b^	0.01	0.1 (0.1)^a^	0.2 (0.1)^a^	0.1 (0.1)^a^	0.70
Change during COVID	0.1 (0.2)	−0.0 (0.2)^a^	−0.0 (0.2)^a^	0.60	−0.0 (0.2)^a^	0.1 (0.2)^a^	0.0 (0.2)^a^	0.61
Eat foods that are traditionally purchased pre-made but someone made at home^5^								
Before COVID	1.4 (0.2)^a^	1.5 (0.2)^a^	1.4 (0.2)^a^	0.07	1.4 (0.2)^a^	1.4 (0.2)^a^	1.5 (0.2)^a^	0.16
Change during COVID	0.1 (0.1)^a^	0.1 (0.1)^a^	0.3 (0.1)^b^	<0.0001	0.2 (0.1)^a^	0.1 (0.1)^b^	0.2 (0.1)^ab^	0.003
Eat something from a fast-food restaurant^4^								
Before COVID	2.7 (0.3)^a^	2.8 (0.3)^a^	2.7 (0.3)^a^	0.07	2.7 (0.3)^a^	3.0 (0.3)^b^	2.5 (0.3)^a^	0.0002
Change during COVID	−0.3 (0.3)^a^	−0.6 (0.3)^a^	−0.7 (0.3)^a^	0.21	−0.6 (0.3)^a^	−0.6 (0.3)^a^	−0.4 (0.3)^a^	0.16
Eat takeout or delivery from a restaurant^4^								
Before COVID	2.3 (0.2)^a^	2.3 (0.3)^a^	2.3 (0.3)^a^	0.39	2.3 (0.3)^a^	2.6 (0.3)^b^	2.1 (0.3)^a^	<0.0001
Change during COVID	−0.2 (0.3)^a^	−0.2 (0.3)^a^	−0.2 (0.3)^a^	0.28	−0.1 (0.3)^a^	−0.4 (0.3)^b^	−0.1 (0.3)^ab^	0.02
Eat at a restaurant either indoors or outdoor dining^4^								
Before COVID	2.2 (0.2)^a^	2.5 (0.2)^b^	2.7 (0.2)^c^	<0.0001	2.5 (0.2)^a^	2.4 (0.2)^a^	2.5 (0.2)^a^	0.16
Change during COVID	−1.3 (0.2)^a^	−1.6 (0.2)^b^	−1.9 (0.2)^c^	<0.0001	−1.6 (0.2)^a^	−1.5 (0.2)^a^	−1.7 (0.2)^a^	.16
Eat meals that are home-cooked^4^								
Before COVID	7.8 (0.7)^a^	7.8 (0.7)^a^	7.5 (0.7)^a^	0.50	8.1 (0.7)^a^	6.9 (0.7)^b^	8.0 (0.7)^a^	0.0005
Change during COVID	0.4 (0.5)^a^	0.8 (0.5)^ab^	1.2 (0.5)^b^	0.0001	0.8 (0.5)^a^	0.8 (0.5)^a^	0.8 (0.5)^a^	0.68
Eat pre-packaged meals such as frozen dinners, canned soup, or ramen noodles^4^								
Before COVID	3.3 (0.4)^a^	2.5 (0.4)^b^	2.2 (0.4)^b^	<0.0001	2.5 (0.4)^a^	2.4 (0.4)^a^	3.1 (0.4)^b^	<0.0001
Change during COVID	0.2 (0.3)^a^	0.0 (0.3)^a^	0.0 (0.3)^a^	0.44	0.1 (0.3)^a^	0.0 (0.3)^a^	0.1 (0.3)^a^	0.89
Throw food away								
Before COVID	2.6 (0.1)^a^	2.6 (0.1)^a^	2.6 (0.1)^a^	0.61	2.7 (0.1)^a^	2.5 (0.1)^b^	2.7 (0.1)^a^	0.0001
Change during COVID	0.1 (0.1)^a^	0.1 (0.1)^a^	0.1 (0.1)^a^	0.17	0.2 (0.1)^a^	0.1 (0.1)^a^	0.1 (0.1)^a^	0.09

*Note*: Sustainable diet means with common superscript letters did not differ at *p* < 0.05 using Tukey’s Studentized Range.

1 Adjusted for education, race, and gender.

2 Adjusted for education, ITN, and gender.

3 0-4, where 4 = Always and 0 = Never.

4 Times per week.

5 0-4, where 0 = Always and 4 = Never.

High-income and Black/African American participants reported the greatest increases in the human health dimension of sustainable diets during the pandemic. With regard to income, these changes were strongly driven by high-income participants eating more fish (0.1 (0.2)) during the COVID-19 pandemic as compared to before the COVID-19 pandemic (Table 6). Meanwhile, Black/African American participants’ increases in the human health dimension were due to consuming more vegetables (0.9 (0.2)), fruit (0.8 (0.1)), and fish (0.2 (0.2)), and fewer sweets (0.3 (0.2)), than before the COVID-19 pandemic.

**Table 6.** Associations between income, race/ethnicity and human health dimension (n = 1,001)

	Household Income^1^			P-value	Race/Ethnicity^2^			P-value
	Low	Moderate	High		Hispanic or Latino	Black or African American	White	
	Mean (SE)				Mean (SE)			
Change in human health dimension before and during COVID								
Vegetables^3^	0.7 (0.2)^a^	0.6 (0.2)^a^	0.7 (0.2)^a^	0.49	0.6 (0.2)^a^	0.9 (0.2)^b^	0.5 (0.2)^a^	<0.0001
Fruit^3^	0.6 (0.1)^a^	0.5 (0.2)^a^	0.6 (0.2)^a^	0.11	0.6 (0.2)^a^	0.8 (0.1)^b^	0.4 (0.2)^c^	<0.0001
Nuts^3^	−0.2 (0.1)^a^	−0.1 (0.1)^ab^	−0.1 (0.1)^b^	0.02	−0.2 (0.1)^a^	−0.1 (0.1)^a^	−0.1 (0.1)^a^	0.86
Beef, lamb, or pork^4^	0.4 (0.1)^a^	0.4 (0.1)^a^	0.4 (0.1)^a^	0.90	0.5 (0.1)^a^	0.4 (0.1)^a^	0.3 (0.1)^a^	0.11
Potatoes^4^	−0.1 (0.2)^a^	−0.1 (0.2)^a^	−0.0 (0.2)^a^	0.20	−0.1 (0.2)^a^	−0.0(0.2)^a^	−0.1 (0.2)^a^	0.61
Fish^3^	−0.2 (0.2)^a^	0.0 (0.2)^b^	0.1 (0.2)^b^	0.0001	−0.1 (0.2)^a^	0.2 (0.2)^b^	−0.1 (0.2)^a^	0.0002
Beans, lentils, or peas^3^	−0.2 (0.1)^a^	−0.1 (0.1)^ab^	−0.1 (0.1)^b^	0.003	−0.1 (0.1)^a^	−0.2 (0.1)^a^	−0.1 (0.1)^a^	0.16
Dairy^4^	0.2 (0.1)^a^	0.1 (0.1)^a^	0.1 (0.1)^a^	0.83	0.1 (0.1)^ab^	0.3 (0.1)^a^	0.1 (0.1)^b^	0.02
Whole grains^3^	−0.1 (0.1)^a^	−0.1 (0.1)^a^	−0.0 (0.1)^a^	0.08	−0.0 (0.1)^a^	−0.0 (0.1)^a^	−0.1 (0.1)^a^	0.11
Sweets^4^	0.2 (0.2)^a^	0.1 (0.2)^a^	0.2 (0.2)^a^	0.22	0.1 (0.2)^ab^	0.3 (0.2)^a^	0.1 (0.2)^b^	0.02
Eggs^4^	−0.2 (0.1)^a^	−0.2 (0.2)^a^	−0.4 (0.2)^b^	0.0005	−0.4 (0.1)^a^	−0.1 (0.1)^b^	−0.3 (0.1)^a^	<0.0001
Chicken and other poultry^4^	−0.4 (0.1)^ab^	−0.4 (0.1)^a^	−0.5 (0.1)^b^	0.03	−0.5 (0.1)^a^	−0.5 (0.1)^a^	−0.4 (0.1)^a^	0.24
Soy food (including tofu and soy milk)^3^	−0.4 (0.2)^a^	−0.2 (0.2)^b^	−0.2 (0.2)^b^	<0.0001	−0.2 (0.2)^a^	−0.4 (0.2)^b^	−0.2 (0.2)^a^	0.005

*Note*: Sustainable diet means with common superscript letters did not differ at *p* < 0.05 using Tukey’s Studentized Range.

1 Adjusted for education, race, and gender; analysis was conducted using proc glm lsmeans.

2 Adjusted for education, ITN, and gender; analysis was conducted using proc glm lsmeans.

3 -2 to 2, where -2 = A lot less and 2 = A lot more.

4 -2 to 2, where -2 = A lot more and 2 = A lot less.

## Discussion

The goal of this study was to describe changes among US adults’ ecological, economic, human health, socio-cultural, and political dimensions of a sustainable diet within the Michigan food environment one year into the COVID-19 pandemic. Our hypothesis that behaviours became on average more supportive of a sustainable diet one year into the COVID-19 pandemic compared to before the COVID-19 pandemic was supported by study findings with regard to ecological, economic, and human health dimensions of sustainable diets. In March 2021 COVID-19 restrictions were beginning to ease in Michigan but were still widely enforced.^(32)^ In regard to the food environment, restaurant capacity was only allowed to resume to 50% up to 100 people, tables needed to be 6 feet apart, and an 11 pm curfew was in place.^(32)^ Additionally, residential indoor gatherings were permitted up to 15 people and outdoor gatherings were allowed up to 50 people.^(32)^ As such, gains within the ecological and economic domain were largely likely due to social distancing and strict prohibitions on indoor dining in Michigan, which resulted in participants eating at restaurants less frequently and eating home-cooked meals more frequently one year into the COVID-19 pandemic compared to before.^(7,8)^ Furthermore, shelter-in-place and social distancing policies also likely contributed to participants driving to the grocery store less frequently during the COVID-19 pandemic and increasing home delivery of groceries. These changes may have decreased GHGE from fuel use.^(33)^ Additionally, the human health dimension of sustainable diets improved through increased consumption of vegetables, fruits, and nuts, and eating less meat during the COVID-19 pandemic as compared to before.

While many of the improvements observed were due to policy changes that do not make sense outside of a pandemic, this study shows that consumer behaviour is modifiable and there are changes to policies and environments that could help consumers maintain these more sustainable behaviours. In particular, less frequent trips to the grocery store and increased use of home grocery delivery options could be targeted. In the US where most consumers drive to the grocery store, having groceries delivered to the home is on average more sustainable;^(33)^ however, it is important to note that the overall frequency of groceries acquired (when done so in a car or van) has a larger impact than delivery method.^(14)^ Therefore, home delivery options could use a minimum item order requirement or a fiscal incentive to lessen the frequency of deliveries per customer. As the risk associated with the COVID-19 pandemic has lessened, consumers have begun shopping again more frequently in person.^(34)^ As such, increased access to public transportation would be a way to incentivize consumers to continue taking fewer trips in their cars to the grocery store. In countries where residents regularly drive to the store, walking or biking instead would result in even lower GHGE compared to online delivery shopping.^(33)^ Consequently, improving access to public transportation (sidewalks, bike lanes, buses, and trains) in urban and peri-urban areas throughout the US could facilitate even greater improvements to the sustainability of acquiring food than having groceries delivered to individuals’ homes. Still, the overall proportion of food’s carbon footprint that is accounted for by transportation is small. So while reducing food miles is useful to consider, it is not the core issue to be addressed with respect to food-related GHGEs.

Another positive change that occurred during the COVID-19 pandemic was an increase in eating home-cooked meals along with greater consumption of vegetables, fruits, and nuts. It is important to encourage the continuation of these habits as they are not only better for the ecological and economic dimensions of a sustainable diet (due to greater alignment with a plant-based diet)^(35)^ but could also help to support the positive changes to the human health dimension observed in study participants mentioned above.^(36)^ Lack of time and cooking skills are often-cited obstacles to cooking at home.^(37)^ Not having enough time to cook could be addressed by reducing the amount of time employees spend commuting each day to and from work. Some companies have offered the continuation of work-from-home options that were required during the early months of the COVID-19 pandemic.^(38)^ The United States Department of Agriculture (USDA), Economic Research Service found that the time employees spend cooking and the number of meals eaten at home is greater when people work from home compared to working in person.^(39)^ However, this is not an option for all occupations, especially for low-income service jobs.^(38)^ Other ways to minimise the time required to cook at home are by cooking food in bulk for the week and portioning food into meals.^(40)^ Overall, it is important to help support the positive changes to ecological, economic, and human health dimensions of a sustainable diet with a diverse suite of interventions.

When assessed through the socio-cultural and political dimension, the overall improvements in sustainable diet dimensions during the COVID-19 pandemic were not equitably experienced across socioeconomic groups. For example, low-income participants reported significantly fewer improvements in the human health dimension than higher-income participants. These differences can largely be explained by high-income households increasing their fish consumption, while in low-income households it declined. The increase among higher-income individuals may have been an approach to maintain protein intake as a result of decreased beef, lamb, and pork consumption. Decreased consumption of these animal proteins was seen among all income levels, likely due to disruptions in the meat supply chain which resulted in higher prices and lower availability.^(41)^ There is, however, marginal room for Americans overall to increase their fish consumption while staying within sustainable parameters.^(10)^ Therefore, in order for low-income households to have improved sustainable diets, alternative sources of protein must be explored, and plant-based proteins such as legumes are a sustainable, low-cost option.^(42)^ Unfortunately, in our study population, low-income households ate fewer beans and soy during the COVID-19 pandemic than their high-income counterparts. Plant-based proteins could be promoted to low-income families at the grocery store through the addition of certain types of plant-based proteins in the Double Up Food Bucks programme (e.g., beans, peas, lentils, and other minimally processed legumes). Double Up Food Bucks currently offers lower-income people who have an EBT/Bridge Card or are on SNAP the opportunity to match their fruit and vegetable purchases up to $20 per day.^(43)^ This intervention could increase low-income households’ access to and consumption of legumes. However, it is important to acknowledge that legume consumption is not part of the typical US dietary pattern albeit this varies by cultural preference and ethnicity which could provide additional barriers to increasing legume consumption.^(44)^

Further differences in sustainable diets were observed within the socio-cultural and political dimensions by race/ethnicity. A notable difference across different racial/ethnic groups was the change in the human health dimension of sustainable diets during the COVID-19 pandemic. When adjusting for household income, White participants reported few changes in the human health dimension during the COVID-19 pandemic while Black/African American participants increased their human health dimension score substantially through increased intake of vegetables, fruit, and fish, as well as decreased intake of sweets. These changes may be due to the fact that Black or African American people were disproportionately affected by the COVID-19 pandemic and may have been shifting their behaviour to improve their nutrition as a way to prevent falling severely ill from COVID-19.^(45,46)^ To address the inequities at the root of the pandemic’s greater impact on Black and African American communities, future interventions for dietary change may need to grapple with structural racism in the US food environment,^(47)^ as it is in part responsible for nutrition and health inequities, including those that widened during the COVID-19 pandemic.

It is worth highlighting one change that occurred across all races/ethnicities: a decline, of about 50%, in local food system engagement (farmer’s markets and CSAs). This decline in community engagement likely occurred in response to a change in the broader food environment strongly mediated by restrictions on public gatherings. However, unlike grocery stores that may have offered online shopping or home delivery options, many farmers were unable to provide consumers with these options due to a lack of (access to) technical resources.^(48)^ Some farmers and local food organisations were able to make the transition to contact-free shopping, developing innovative systems that allowed local farmers to sell to consumers outside of traditional farmer’s markets and CSAs (e.g., farm-to-home delivery and online purchasing options), and thereby increasing access to sustainable food options during the pandemic.^(48)^ Similar systems could be implemented by farmer’s markets and CSAs to strengthen the resilience of local food systems.

A noteworthy strength of this study is its large, sociodemographically diverse population-based sample with respect to income and race/ethnicity. However, as Black/African American, Hispanic/Latino, and White individuals were specifically recruited, this study did not capture the experience of other racial or ethnic groups and findings cannot be generalised to these populations. Furthermore, data were collected using convenience sampling, a form of non-probability sampling that relies on self-selected participation. Our sample also consisted of people who received Outpatient care at Michigan Medicine. This approach excludes people who did not seek care and may have over-sampled healthier people who seek primary care than the general population.^(49)^ The study relied on participants’ recall and therefore the potential for recall bias is possible. In particular, since the survey asked participants to recall their behaviour at two different points in time, participants may be better able to recall the more recent period (over the past year during the COVID-19 pandemic) than before the COVID-19 pandemic. Our study did not collect information regarding participants’ health status, so we are not able to determine if, or to what extent, this occurred. Recruitment also required that participants had private or public health insurance and thereby would exclude the uninsured population. Additionally, this study required that participants had an email address in their medical record. Participant responses may also have been impacted by social-desirability bias, particularly given the highly politically polarised nature of social distancing protocols that were put into place to diminish the spread of COVID-19. To mitigate social-desirability bias, survey response data was collected online, and minimal personally identifiable data was collected to maintain anonymity.

Overall, adults engaged in more behaviours that are supportive of a sustainable diet during the COVID-19 pandemic as compared to before the COVID-19 pandemic. This is particularly true regarding ecological and economically sustainable behaviours including fewer trips to the grocery store, increased use of home grocery delivery options, increased cooking at home, and greater consumption of healthy foods, such as vegetables and fruits. In order to support the continuation of these behaviours beyond the context of a global pandemic, policies that increase access to public transportation, limit the frequency with which consumers have groceries delivered, and increase work-from-home options should be supported. Not all behaviour changes during the pandemic were positive with respect to sustainable diets as local food system engagement declined. These declines specifically highlight the importance of supporting local farmers and food systems to strengthen their resilience to consumers’ changing needs. Additionally, this study found that the food environment in Michigan during the COVID-19 pandemic appears to have been changed in differing ways for different socioeconomic and racial/ethnic groups in regard to sustainable diets. In order to address these issues of equity, multiple public health policies and interventions are needed to increase access to sustainable diets. Future research should reassess the ecological and economic, human health, socio-cultural and political dimensions of a sustainable diet to see what, if any changes have been maintained long-term.

## Supporting information

Ludwig-Borycz et al. supplementary material Ludwig-Borycz et al. supplementary material

## Supplementary material

The supplementary material for this article can be found at https://doi.org/10.1017/jns.2024.9
