# Supplementary material for: Changing sustainable diet behaviours during the COVID-19 Pandemic: inequitable outcomes across a sociodemographically diverse sample of adults
Source: J Nutr Sci. 2024 Mar 14;13:e16. doi: 10.1017/jns.2024.9 (PMC10988137; doi:10.1017/jns.2024.9)
Supplement: Ludwig-Borycz et al. supplementary material [file S2048679024000090sup001.docx]

Participants who completed screening survey **n=2,625**

Participants who did not meet eligibility criteria (living in the state of Michigan since at least March 2020, involved in food choices/shopping for their household, ages 18 through 65 years old, and fluent in English) **n=186**

Participants eligible to participate **n=2,439**

Excluded because enrollment limits had already been met **n=951**

Participants completed study survey **Table 1 and 2 n=1,488**

Participants with race/ethnicity other than White, Black, or Hispanic; missing vales for ITN; or missing values for covariates were excluded **(n=142)** for the difference in means analyses **n=1,346**

**Supplemental Figure 1. SUSTAIN Study Participant Flow Chart**

**Supplemental Table 1. Ecological and Economic Sustainable Diet Behaviors Queried in the SUSTAIN Survey**.

| **Behaviors** | **Response Options** |
| --- | --- |
| 1. Drive to the grocery store 2. Have groceries delivered to your home | Never or less than once a month |
|  | 1-2 times per month |
|  | 3-4 times per month |
|  | 2-3 times per week |
|  | 1 or more times per day |
| 1. Shop for locally grown produce and/or other food 2. Shop at a farmer’s market or participate in a CSA (Community Supported Agriculture) 3. Grow a vegetable garden or participate in a community garden 4. Eat foods that someone made at home, that are traditionally purchased pre-made (e.g. bread, muffins, or granola) | Never |
|  | Rarely |
|  | Sometimes |
|  | Frequently |
|  | Always |
| 1. Eat something from the following types of restaurants:    1. fast food (include traditional “burgers-and-fries,” Mexican, fried chicken, sandwich or sub shop, and pizza)    2. eat at a restaurant in person    3. eat take-out/delivery from a restaurant 2. Eat food that is cooked at home 3. Eat pre-packaged/ ready-made meals 4. Order a meal kit delivery | Never or less than one meal per month |
|  | 1-4 meals per month |
|  | 2-6 meals per week |
|  | 1 meal per day |
|  | 2 or more meals per day |
| 1. Throw away foods (e.g. vegetables, fruit, meat, poultry, fish, and grains) | None |
|  | Very little |
|  | Little |
|  | Some |
|  | Much |
